# Delivering patient decision aids on the Internet: definitions, theories, current evidence, and emerging research areas

**DOI:** 10.1186/1472-6947-13-S2-S13

**Published:** 2013-11-29

**Authors:** Aubri S Hoffman, Robert J Volk, Anton Saarimaki, Christine Stirling, Linda C Li, Martin Härter, Geetanjali R Kamath, Hilary Llewellyn-Thomas

**Affiliations:** 1 The Dartmouth Institute for Health Policy and Clinical Practice, Geisel School of Medicine at Dartmouth College, 46 Centerra Parkway (HB7250), Lebanon, New Hampshire 03766, USA; 2 Department of General Internal Medicine, Unit 1465, The University of Texas MD Anderson Cancer Center, 1515 Holcombe Blvd, Houston, Texas 77230, USA; 3 Clinical Epidemiology Program, Ottawa Hospital Research Institute, 501 Smyth Road, Ottawa, Ontario K1H 8L6, Canada; 4 School of Nursing and Midwifery, University of Tasmania, 17 Booker Avenue, Hobart, Tasmania, Australia 7000; 5 Department of Physical Therapy, University of British Columbia, Arthritis Research Centre of Canada, 5591 No. 3 Road, Richmond, British Columbia V6X 2C7, Canada; 6 Department of Medical Psychology, University Medical Center Hamburg-Eppendorf, W26, Martinistraße 52, 20246 Hamburg, Germany; 7 Department of Community and Family Medicine, The Geisel School of Medicine at Dartmouth, Hanover, NH 03755, USA; 8 The Dartmouth Institute for Health Policy and Clinical Practice, The Geisel School of Medicine at Dartmouth, Hanover, NH 03755, USA

## Abstract

**Background:**

In 2005, the International Patient Decision Aids Standards Collaboration identified twelve quality dimensions to guide assessment of patient decision aids. One dimension—the delivery of patient decision aids on the Internet—is relevant when the Internet is used to provide some or all components of a patient decision aid. Building on the original background chapter, this paper provides an updated definition for this dimension, outlines a theoretical rationale, describes current evidence, and discusses emerging research areas.

**Methods:**

An international, multidisciplinary panel of authors examined the relevant theoretical literature and empirical evidence through 2012.

**Results:**

The updated definition distinguishes Internet-delivery of patient decision aids from online health information and clinical practice guidelines. Theories in cognitive psychology, decision psychology, communication, and education support the value of Internet features for providing interactive information and deliberative support. Dissemination and implementation theories support Internet-delivery for providing the right information (rapidly updated), to the right person (tailored), at the right time (the appropriate point in the decision making process). Additional efforts are needed to integrate the theoretical rationale and empirical evidence from health technology perspectives, such as consumer health informatics, user experience design, and human-computer interaction.

Despite Internet usage ranging from 74% to 85% in developed countries and 80% of users searching for health information, it is unknown how many individuals specifically seek patient decision aids on the Internet. Among the 86 randomized controlled trials in the 2011 Cochrane Collaboration’s review of patient decision aids, only four studies focused on Internet-delivery. Given the limited number of published studies, this paper particularly focused on identifying gaps in the empirical evidence base and identifying emerging areas of research.

**Conclusions:**

As of 2012, the updated theoretical rationale and emerging evidence suggest potential benefits to delivering patient decision aids on the Internet. However, additional research is needed to identify best practices and quality metrics for Internet-based development, evaluation, and dissemination, particularly in the areas of interactivity, multimedia components, socially-generated information, and implementation strategies.

## Background

In 2005, the International Patient Decision Aids Standards (IPDAS) Collaboration identified twelve dimensions that could guide the assessment of the quality of patient decision aids (PtDAs) [1,2]. One of these dimensions—the delivery of PtDAs on the Internet—is relevant when the Internet is used to provide some or all components of a PtDA. This dimension raises a unique challenge in assessing quality, requiring an integration of relevant definitions, theories, and evidence from decision science, medicine, medical/consumer health informatics, user experience design, human-computer interaction, health services research, and implementation science. Therefore, the aims of this paper are to provide an updated definition for this dimension, outline the current theoretical rationale for Internet-delivery as an important aspect of quality assessment, identify the gaps in empirical evidence, and discuss emerging research areas needed for assessing quality.

## An updated definition

“Delivering patient decision aids on the Internet” is defined as the process of using the Internet to provide some or all components of a PtDA to help individuals (e.g., patients, caregivers, proxy decision makers, etc.) involved in the process of choosing between two or more medically-appropriate healthcare options (e.g., preference-sensitive care). This definition is intended to differentiate Internet-delivered PtDAs that support shared decision making in preference-sensitive care from online health education websites, clinical practice guidelines, clinicians’ decision support, or expert systems [3].

This broad conceptual definition covers a range of operational approaches—from providing a downloadable copy of a paper-based PtDA, to streaming a video-based PtDA online, to providing interactive decision support websites that tailor information and support to the needs and preferences of each individual decision maker. It is important to note that delivery of a PtDA on the Internet may be only one component of a larger decision support program (e.g., viewing a PtDA website coupled with a phone call from a decision coach). Internet-delivered PtDAs may also be used during, in conjunction with, or separate from a clinical consultation. However, the purpose of this definition is to provide a perspective from which patients, families, clinicians, decision aid developers, and policy makers can evaluate the quality of the manner in which a PtDAs is delivered on the Internet.

## Updated theoretical justification

The current rationale for delivering PtDAs on the Internet draws on a range of disciplines, including psychology, education, and implementation science. Several theories in cognitive psychology, decision psychology, and communication emphasize the value of using the Internet to provide broad and long-term dissemination of information that can be targeted and tailored to patients’ needs and preferences. For example, the Health Belief Model emphasizes the importance of providing tailored information to motivate active engagement in health care [4]. Similarly, interactive deliberative tasks foster self-efficacy and lead to increased engagement (Social Cognitive Theory) [5]. The Elaboration Likelihood Model proposes that people attend to and actively process information more if it is perceived as personally-relevant [6]. The Theory of Goal Setting and Performance supports the role of interactivity in producing tailored actionable personal health goals [7]. Finally, the Stages of Change Theory supports the value of having up-to-date information and support readily accessible over time, so that patients can revisit the information and support across the progression of disease and multiple iterative decision-making cycles [8].

From the perspective of the individual patient, theories of active learning, discovery learning, and social learning inform the design of Internet-delivered decision support tools that effectively inform and educate [9]. Behaviorism emphasizes the need for measurable behaviors to confirm learning [10]. Information quizzes can reinforce awareness and facilitate realistic expectations of outcomes. Cognitive psychology emphasizes the internal processes of memory, motivation, thinking, and reflection as essential in understanding how new information fits within one’s existing knowledge and experience [11]. Interactive activities such as clarifying one’s personal attitudes about the individual risks and benefits of each treatment option can reinforce comprehension and personalization. Furthermore, constructivism builds on these theories to incorporate observation, processing, and interpretation of information in building one’s personal reality [12]. Ally integrates these three theoretical constructs to describe how health information provided online should be designed to help individuals learn about the “what” (behaviorist), “how” (cognitive psychologist), and “why” (constructivist) of their options [9,13].

At the population level, dissemination and implementation theories emphasize the importance of establishing effectiveness for the maximum number of people. Theories of innovation, knowledge translation, and organizational change may inform strategies for effective use of web-based PtDAs within medical centers and associated clinics [14,15]. Health equity frameworks support the development of technology-supported interventions to extend the reach of medical centers into rural or underserved communities. Community organizing models such as Reach Effectiveness Adoption Implementation (RE-AIM), Precede-Proceed, etc., can guide consumer-informed design approaches, particularly for chronic disease self-management and for implementation within community programs [16,17].

## Updated empirical evidence

The theoretical justification outlined above supports the idea of using the Internet to deliver PtDAs, although there are notable gaps in the empirical evidence. Three primary barriers must be addressed before a comprehensive systematic review of the effectiveness and quality of Internet-delivery of PtDAs can be undertaken.

First, greater definitional and methodological clarity is needed. Definitions are needed to distinguish between PtDAs made available for download from the Internet, those adapted for use on the Internet, and those designed and tested as used on the Internet. Efforts are also needed to distinguish among several overlapping terms across disciplines. For example, Internet-delivered PtDAs may be tailored, targeted, customized or personalized (see Additional file 1 Table S1) at several different levels, including: a) at the technology level, if they provide information and technology features based on users’ needs and preferences (consumer health informatics, user experience design) [18]; b) at the decision support level, based on patients’ decision-making needs and preferred deliberative styles (decision science) [3,19]; and c) at the dissemination level, based on cultural, age-related, or decision-making roles (e.g., patient, caregiver, legal proxy, etc.) and membership in user groups (implementation science) [20]. Furthermore, quality metrics may differ by disciplinary perspective; different disciplines focus on assessing the usability of the Internet-delivered tool for supporting patients’ decision making [21], on the usability of features of the user interface [22], and/or on the usability of using Internet delivery to extend patients’ decision support programs into the community [17,22].

Second, empirical studies are needed that specifically assess the need for, and the effectiveness of, Internet-delivery of PtDAs. Since the IPDAS quality criteria were first published [1,2], Internet usage studies have reported continued increase in health information-seeking on the Internet [22-28], but have not specifically assessed the usage rates for PtDAs. Online libraries of PtDAs provide access to an increasing number of PtDAs [29], but few PtDAs have been developed using health technology research methods (such as user experience design, human computer interaction, etc.) or have been evaluated for effectiveness as used on the Internet. A Cochrane Collaboration review of randomized controlled trials of the effectiveness of PtDAs began in 1996, and was recently updated in 2011 to include trials published through December 2009 [30]. However, Internet-delivery of PtDAs was not the focus of that review. Accordingly, a comprehensive systematic review of decision science, health technology, and implementation science literature specifically focusing on the Internet-delivery of PtDAs is needed.

Lastly, since Internet-delivery involves both human and computer factors, further empirical evidence is needed from multiple disciplinary perspectives. From the human factors perspective, the recent growth in making PtDAs available on the Internet has provided some evidence from the fields of decision psychology, health education, and medicine. Studies are emerging regarding the development and/or field testing of new Internet-delivered PtDAs, but, at this point, few randomized controlled trials of effectiveness have been completed. From the Internet-delivery perspective, evidence from information technology perspectives—such as medical/consumer health informatics, user experience design, and human-computer interaction— need to be applied and tested in the context of patient decision support. For example, some online PtDAs may produce personalized risk estimates based on patients’ clinical profiles (as in the field of medical informatics). Others may gather patients’ reported informed, values-based preferences into health information systems (as in the field of consumer health informatics). Furthermore, attention must be paid to human-computer interaction and evidence about user experiences with design must be gathered, in order not only to identify “best practices” in the design of Internet-based PtDAs but also to inform quality assessment measures and guidelines. At present, any attempt to carry out a full systematic review of Internet-based PtDAs is limited by these three barriers; accordingly, the following sections at least attempt to outline what is currently known and to highlight the gaps in empirical evidence regarding Internet-delivery of PtDAs.

### Use of the Internet for decision support

For 2007-2011, http://Worldbank.org indicates that an average of 74.2% of individuals in the United States, 76% in Australia, 81% in Canada, 83% in Germany, and 85% in the United Kingdom use the Internet [23]. In 2011, the worldwide population of Internet users exceeded 3 billion (35% of the 7 billion world population), with over 1.8 billion homes receiving direct Internet access. In developing countries, 25% of individuals have a computer, and 20% have Internet access. A 2011 survey in the United States [24] indicated that 80% of Internet users search for health information, making it the third most popular online activity (behind using e-mail and using search engines). Most of these individuals search for information about a specific disease (66%) or treatment procedure (56%).

Similarly, the 2005-2010 International Telecommunication Union estimates mobile phone usage rates at 90% of individuals in Korea, 83% in Australia, 75% in China, 59% in Italy, 56% in the United Kingdom, 38% in the Netherlands, and 30% in Canada [26]. By 2011, the number of mobile-broadband subscriptions increased to 1.2 billion worldwide, with 90% of those subscriptions covered by 2G mobile-cellular networks and 45% covered by 3G networks. Studies by the Pew Internet & American Life Project [27] reported a continuation in the upward trend in mobile phone usage in the U.S. Over 83% of U.S. adults reported owning a mobile phone, with 35% using a “smart phone” to access the Internet. Mobile Internet usage was most common among individuals who were 18-29 years old, African American, and Latino. Qualitative analyses suggested a growing preference for social connectivity (e.g., high rates of texting and social websites), and for searching for health information on the Internet prior to making an appointment with a doctor.

In 2007, the DECISIONS telephone survey asked patients about their memory of using the Internet for nine selected medical decisions within the past two years [28]. The survey sample consisted of English-speaking U.S. adults (N = 2575) who, within the last two years, had talked with a clinician about, or had undergone, a procedure for one of nine decisions about medications (high blood pressure, high cholesterol, depression), screening (colorectal, breast, prostate), or surgery (cataracts, lower back pain, hip/knee replacement). An average of 28% of participants self-reported using the Internet related to these nine health decisions within the previous two years. Percentages varied from 17% for breast cancer to 48% for hip/knee replacement. Internet use decreased with age and increased by income level. Participants rated the Internet as their second-most important information source (their clinician being the first). Taken together, these studies confirm the continued rise in rates of Internet use, health information-seeking, and Internet searching related to medical decisions. However, due to the lack of clarity in terminology and the limited assessment efforts, statistics regarding how many individuals seek PtDAs on the Internet remain unknown.

### Inventories of PtDAs delivered on the Internet

Multiple inventories of PtDAs are available on the Internet, but “Internet-delivery” is currently operationalized in several ways. For example, the Ottawa Hospital Research Institute maintains a Patient Decision Aid Library Inventory that provides browseable lists of PtDAs by clinical topic, as well as contact information and IPDAS quality scores [29]. As of October 2012, this inventory contained 270 PtDAs, 198 of which were available in some form on the Internet. Currently, most of these PtDAs are available as brochures, worksheets, or videos that patients can download and use offline. Some have been adapted for viewing online. More recently, a few have been specifically designed for interactive use on the Internet. These differences may represent sub-categories of Internet delivery, for which different evaluative measures are appropriate to assess their quality as used on the Internet (see Tables 1, 2 and Additional file 1 Table S1).

**Table 1:** Development Characteristics of PtDAs Delivered on the Internet [27-30,72,73]

**Internet-delivered PtDAs**	Includes all PtDAs for which some or all parts are delivered using the Internet.
**Internet-available PtDAs**	May include PtDAs that were initially developed and tested in other formats (e.g., paper, audio, or video), then made available on the Internet. For example, several PtDA brochures were originally created as paper worksheets, and then made available online for individuals to download, print, and complete.
**Internet-adapted PtDAs**	May include PtDAs created in other formats that were purposefully adapted to allow individuals to use them directly on the Internet. Examples include adapting paper worksheets into interactive questionnaires, and adapting text and video components of PtDA DVDs into websites. While Internet-adapted PtDAs may have been rigorously tested and evaluated in their original format, it is important to consider whether the adapted version has been tested and evaluated as used on the Internet.
**Internet-based PtDAs**	May include PtDAs that were specifically designed and tested for use on the Internet. Examples include websites designed to help patients with specific health care decisions by interactively tailoring information and support to their needs, or by providing opportunities for family members to participate in the discussion. Internet-based features may also include e-mail, discussion forums, blogs, or social media sites (e.g., Facebook, patient community websites) facilitated by clinicians, decision coaches, peers, or patient advocacy groups.

**Table 2:** Evaluative Characteristics of PtDAs Delivered on the Internet [20,33-43]

**Accessibility of the Technology**	Refers to the degree to which all people can access the Internet using whichever device they prefer (e.g., computer, laptop, tablet, or mobile phone) regardless of available dial-up/high-speed, Wi-Fi, or mobile phone Internet service. Dissemination strategies to maximize technology accessibility include providing both text-heavy and graphics-heavy versions of a PtDA, as well as versions for multiple screen sizes and mobile phones (known as *responsive design*).
**Universality of the Technology**	Refers to the degree to which the PtDA is accessible for men and women of all ages, races, ethnicities, religions, languages, and cultures.
**Usability of the Technology**	“…refers to how well users can learn and use a product to achieve their goals and how satisfied they are with that process” (ISO/AWI TR 9241-11, 1998) [43]. This definition includes a combination of factors, primarily focusing on five areas: 1. *Ease of Learning* - How easy it is to do basic tasks the first time you see the website, 2. *Efficiency of Use* - How quickly you can use the website once you know how, 3. *Memorability* - How well you can remember how to use it the next time you visit, 4. *Error Frequency* - How many errors are typically made in looking for information, and 5. *Satisfaction* - How much you like using the website.

### Studies of Internet delivery of patient decision aids

While a systematic review of Internet delivery of PtDAs remains to be done, the 2011 update of Cochrane Collaboration’s review of the effectiveness of PtDAs includes four initial randomized controlled trials that used the Internet.[30] This systematic review included randomized controlled trials that a) were published up to December 2009, b) compared a PtDA with usual care or an alternative intervention, and c) involved individuals who were actively making a treatment or screening decision. Among the 86 studies that met these criteria, four studies tested Internet delivery approaches. Table 3 provides a summary of each study’s clinical topic, study design, and primary results.

**Table 3:** Most Recent (2011) Cochrane Collaboration Review of PtDAs [30]: Randomized Controlled Trials that Involved Internet Delivery [72-75]

Author, Year, Reference Number	Clinical Context	Study Design	Primary Results
Frosch, 2003, [72]	PSA screening	RCT, n = 226 randomized to view video during appointment at clinic or website at home before clinic visit	For both groups: - Knowledge scores were similar; - Ratings of effort required, convenience, and satisfaction were similar. Viewing the video at the clinic: - Increased the likelihood of viewing the complete PtDA
Ruffin, 2007, [74]	Colorectal Web, cancer screening	RCT, n = 174 men randomized to either informational website or Colorectal Web	Viewing Colorectal Web: - increased immediate reporting of preferred test, but no difference at 2, 8, or 24 weeks - increased screening
Krist, 2007, [75]	PSA screening	Clustered RCT, n = 497 randomized to paper PtDA, website PtDA, or no pre-visit education	Viewing either PtDA: - Increased decisional control - Increased knowledge scores - Decreased screening
Frosch, 2008, [73]	PSA screening	RCT, n = 611 randomized to web-based didactic PtDA, disease model + time trade-off exercise, both, or public PSA websites	Knowledge scores were: - Highest for didactic PtDA - Lowest for public websites Post-PtDA screening preferences differed across groups.

Results of these four randomized controlled trials indicate that using an Internet-delivered PtDA: a) improved knowledge scores; b) was rated similarly on effort needed, convenience, and satisfaction; and c) had variable effects on preferences for screening. While these studies are the first randomized controlled trials to include Internet-delivery, initial results must be interpreted cautiously. Potential confounding variables were not addressed explicitly in the study reports, such as whether patients were involved in initial decision support and technology needs assessments [19] prior to the development of the Internet-delivered version; whether usability/accessibility/field testing (from the perspectives of human computer interaction, user experience design, decision psychology, etc.) had been carried out prior to testing effectiveness; and the possible confounding effects of the selected delivery location and timing relative to the clinical appointment.

A brief review of the literature since 2009 reveals several emerging efforts to develop and test Internet-delivered PtDAs. Additional file 2 Table S2 illustrates a selection of studies from decision support and health informatics publications and presentations at scientific meetings from 2010-2012. Similar to the earlier studies, results indicate that using an Internet-delivered PtDA: a) improved knowledge, preparation for decision making, and decisional conflict scores; b) were acceptable to patients; and c) had variable effects on treatment preferences or the likelihood of receiving screening. These second-generation studies also began the exploration of interactivity and tailoring features, using health informatics and user-centered design methods during development and field-testing. Initial observations indicate that tailoring of clinical information and interactive deliberative guidance may improve decision-making engagement and outcomes.

However, these studies again point to several gaps in the empirical evidence. Additional studies are currently needed to test the role of a) specific Internet features (e.g., audio voice-over, interactive graphics, touch-screen data entry, etc.); and b) different dissemination and implementation strategies (e.g., delivery timing relative to clinic visits, publically-available versus clinician-prescribed; use with/without a decision coach, integration with electronic health records, reach into rural communities, etc.). Subsequent studies may investigate the isolated effect of Internet-delivery of PtDAs when controlled for these factors, as well as the effect of match/mismatch between these factors and patients’ expectations. In order to address these gaps in evidence, multidisciplinary models are needed that include approaches from the fields of informatics, user-centered design, and human-computer interaction. Internet-delivered PtDAs developed without appropriate technology testing (e.g., usability analyses, heuristic evaluations, card sorting, task analyses, etc.) may inadvertently introduce usability biases into the effectiveness trials. Hence, these studies highlight the importance of: a) involving key stakeholders and users in the development and testing of Internet-delivered PtDAs; b) purposefully integrating development and evaluation methods from the fields of health technology, decision support, and implementation science; and c) identifying short- and long-term research priorities.

Lastly, suites of online PtDAs are being rapidly developed by several academic health centers such as Dartmouth College, the Mayo Clinic, and the Universities of Ottawa, Cardiff, Hamburg, Sydney, etc., as well as by private PtDA developers (e.g., Health Dialog, Healthwise, Informed Medical Decision Foundation), health insurers (e.g., Group Health, the U.S. Veterans Administration, Techniker Krankenkasse, etc.), and patient groups (e.g., Alzheimer’s Association, CommonGround, PatientsLikeMe, etc.). As studies led by different disciplines (such as practitioners in decision support, health informatics, business leadership) and from perspectives (e.g., academic, governmental, private, and patient-led) converge, evidence may be systematically reviewed and critically assessed to generate multidisciplinary, evidence-based models, methods, and quality measures.

## Discussion

Building on the updated definition, theoretical rationale, and empirical evidence, the following discussion highlights some emerging areas for research that can contribute towards developing measures and standards of quality for the Internet-delivery of PtDAs.

### Multidisciplinary perspectives on definition

Given the broad definition of “delivering patient decision aids on the Internet”, several subtypes of Internet delivery could be defined. These subtypes could be characterized according to their development, design, and usability characteristics using terms from health technology, decision science, and implementation science (see Tables 1, 2 and Additional file 1 Table S1) [22-24,31-43]. It is important to note that many of these terms are currently evolving areas of debate and research (e.g., the relationship between accessibility, universality, and usability), and/or overlap or vary by disciplines (e.g., the usability of the technology versus the usability of the tool within a decision support program). Therefore, these tables are not intended to present an exhaustive list, but may stimulate across-field discussion about terminology and assumptions.

### Theory development

#### Social connectivity and patients’ stories

Online PtDAs are beginning to include features that allow users to share their stories and to connect virtually for support. These features add to the burgeoning range of distinctive capabilities associated with delivery on the Internet (such as virtual connectivity, interactivity, individualization, real-time evolution of information, socially-derived information, etc.) [31-33], and generate multiple sub-categories across a spectrum of types of online PtDAs. At one end of the spectrum, PtDAs designed using traditional decision support research methods may be adapted online to include patients’ stories in more engaging formats (using, for example, interactive tables or icon arrays that illustrate risks and benefits, and are linked to a patient story for each icon) [44]. They may also add open discussion forums, links to social media sites, or online peer support and guidance in decision making [45]. At the other end of the spectrum, some websites that began as online support groups are now adding decision-making guides and tools for creating individualized risk estimates (e.g., entering one’s personal health information and comparing it against socially-derived averages) [46].

Accordingly, research will need to consider the extent to which established theories (such as Social Development Theory, Elaboration Likelihood Model, etc.) inform the study of how people perceive, value, and use personal experiences that are shared virtually and that evolve in real-time [6,9]. On one hand, some communication and education theories support the use of written or videoed patient stories because their saliency augments the perception of personalized information, and because qualitative studies indicate that patients frequently request such experiential information [33,47,48]. On the other hand, evidence is lacking regarding the potential effects and/or biases that might be introduced by such patient stories. These include, for example: social matching/mismatching between the individuals in the stories and the individuals viewing the decision aid; inadvertent story-induced misrepresentations, such as over/under-weighting of risks/benefits; and hidden story-induced framing biases [49-51]. These effects may be increased or decreased with the further addition of interactivity, virtual social connections, and perceptions of socially-derived “evidence”.

While the presentation of this kind of content carries a high potential for inadvertently presenting biased information to consumers, it also serves as an emerging social experiment highlighting the importance of shared information, connectivity, and the identification of outcomes that may be of the greatest relevance to patients. Further research is needed to clarify: what types of socially-shared content are needed, preferred, and beneficial/harmful to patients; whether this information should be monitored/managed in the context of PtDAs; and whether it can be collected and presented in neutral, unbiased, low literacy, and culturally-relevant formats. Results of such research could, in turn, inform quality metrics related to the incorporation of social interactivity capabilities and socially-derived stories/evidence into Internet-delivered PtDAs.

#### Behavior change

As online PtDAs begin to be used in long-term health behavior change interventions (e.g., when choosing one’s smoking cessation, weight loss, or substance abuse therapies), theories about the stages of behavior change will need to be tested in this new context of Internet-supported decision making. For example, the Trans-Theoretical Model related to Stages of Change has been used in the development of Internet-delivered resources for patients seeking to improve evidence-based healthy behaviors [8], but has not been used for preference-sensitive health care decision making. The role of Internet-delivered deliberative guidance may be more or less effective at specific stages in behavior change, and/or foster more continuous support across stages.

In situations in which decision support is nested in motivational counseling (e.g., when a client first agrees to begin the behavioral change of smoking cessation, and then chooses among available smoking cessation therapies) [52], existing models will need to be tested under conditions in which in-person and online components are mixed or combined. For example, are the patient activation and engagement effects observed during in-person decision support interventions maintained when Internet-delivered PtDAs are nested in virtual counseling interventions (e.g., when using online motivational interviewing, problem solving therapy, or cognitive behavioral therapy)? How will long-term peer support groups be sustained online, and is additional training needed for peer leaders who facilitate these groups? Are additional privacy and security measures needed? Finally, what quality metrics are appropriate in this unique context?

#### Equity

Until Internet access and usage becomes universal, several issues in implementation theory and health equity need to be considered [14,18,20,53]. For example, “direct-to-consumer” delivery of PtDAs on the Internet is currently less feasible for communities that lack either high-speed access or cellular phone service [53]. However, insertion of a telehealth clinic in rural communities can extend health care access for patients who otherwise may be limited by travel times and costs, such as working adults who cannot attend daytime doctor visits, individuals confined to home by chronic medical conditions, and older adults who no longer drive [18]. Furthermore, current trends indicate that retired adults are the fastest growing group of Internet adopters and therefore may benefit from targeted decision support programs on the Internet, but it remains unknown whether this trend is generational or situational (that is, whether the currently-employed generation will also increase their Internet use once they retire) [24]. Research is needed to support the short-, mid-, and long-term prioritization of development strategies for Internet-delivered PtDAs.

In addition, emerging evidence suggests that there are population-wide shifts from personal computers to mobile smart phones, with disadvantaged groups becoming the largest users of smart phones for Internet access [27,28]. Insights gained from research in health informatics and in health communication can provide frameworks and tools for tailoring interventions to a user’s characteristics and preferred Internet access media (e.g., computer, tablet, mobile phone, etc.) [36,54-56]. New efforts are being made to address health equity gaps by developing targeted/tailored mobile health websites and applications [53,57]. These frameworks will need to be applied and tested during the development of PtDAs. Additional research is specifically needed a) to develop methods and quality measures for tailoring according to patients’ preferred deliberative styles (i.e., their preferred level information detail for making decisions, and their favored degree of interactive engagement with decision support), and b) to explore the effects of culturally-tailored experiential information about health care decision making (e.g., socially-derived patient stories). During this transition period in Internet use, theories about implementation and health equity can guide the Internet delivery of PtDAs, while taking into consideration a range of potential short-term and long-term strategies for shifting patient populations and evolving quality-assessment metrics.

#### Chronic illness

There is a need for interdisciplinary frameworks that can guide interventions to address the emerging needs and preferences of those patients with chronic illnesses who engage online with decision support and self-management strategies. Self-management theories support the use of technology for documenting personal experiences over time, and social learning theories support the saliency of shared experiences [33,47]. However, as patients are more frequently reporting their chronic illness experiences on the Internet [46], questions arise about whether and how to incorporate these rapidly-growing banks of patient-reported data into the theoretical frameworks underpinning decision support and evidence-based medicine.

In chronic and progressive illness, decision support involves multiple decision points along the course of disease, multiple delivery settings (e.g., in hospitals, community clinics, nursing homes, senior centers, etc.), and multiple intervention goals (e.g., cure, maintenance, palliation) that may include multiple decision makers (e.g., patient, local caregiver, long distance family members, clinical teams, legal proxies, etc.). Internet delivery offers some unique capabilities that could contribute positively to these complex processes [53,58,59]. For example, a series of related PtDAs can be delivered across the course of a progressive disease. For each decision point, a PtDA may offer support for urgent decision making or can facilitate advanced care planning. Decision support information and activities can be tailored to each user’s role and preferred deliberative style at that point in time. For progressive diseases such as dementia, family members separated by long distances can engage in planning discussions using video chat features and shared family portals. Real-time documentation on decision logs can track progression over time, and could be incorporated into electronic health records.

However, unique challenges also arise—such as creating and maintaining multiple user portals, securing links to electronic health records, and maintaining data over time, etc. [60-62]. In addition, expectations for user-centered design may vary by generation and may need to evolve rapidly alongside advances in direct-to-consumer marketing. Again, an interdisciplinary conceptual framework may be needed to create, maintain, and assess the quality of effective Internet-delivered PtDAs for chronic and progressive conditions.

### Emerging areas in empirical evidence

Interdisciplinary research efforts are also needed to address several gaps in the empirical evidence. These efforts could be considered as gaps in fundamental knowledge, in applied effort, and in research methodology.

#### Fundamental investigations

Fundamental studies conducted by decision scientists may focus on identifying the best methods for operationalizing some or all components of an evidence-based decision support process using the Internet [32,63]. They may also seek to assess whether Internet-delivered PtDAs cause patients to engage differently at various points during their overall decision-making processes. Notably, fundamental research is needed to assess the “value added” of incorporating interactivity, personally-tailored information, interpersonal communication, patient stories, and socially-generated experiential information. Compared to paper- or video-based PtDAs, do the unique features of Internet-based PtDAs (e.g., multimedia, interactive activities, patient portals, social connectivity, etc.) improve patients’ sense of preparation, self-efficacy, and decision quality for the immediate decision, as well as their ability to retain and transfer decision-making skills for future decisions?

Health education scientists may seek to identify which technology features are most helpful for improving information comprehension, which graphic displays best convey risk/benefit information, and which interactive strategies can accurately assess whether a patient is well-informed [64,65]. Cognitive psychologists may investigate whether interactive PtDA websites can foster increased patient activation or increased communication with clinicians, surrogate decision makers, and long-distance family members [59,66,67]. They may also assess which Internet PtDAs stand alone or need to be coupled with personal interaction, and whether the format and features of Internet-based PtDAs should vary by clinical context (e.g., preventive, acute, chronic, or end-of-life treatment decisions).

Consumer health informatics researchers may also face continuing challenges in developing adaptive methods, tools, and measures to respond to the rapidly-changing field of health technologies. What are the emerging technologies and features that are feasible, usable, and sustainable for clinicians, health care systems, and patient advocacy groups that wish to develop Internet-based decision support programs [60-62] Ongoing research will be necessary in order to create tools that are accessible and usable on multiple evolving devices (e.g., computers at the clinic, personal laptops, mobile phones, etc.) Health technology designers also face the challenge of allowing social connectivity and potential public sharing of experiential information for some patients, while ensuring the highest security for the protection of personal health information for others [46,48,60,61].

#### Applied investigations

From the perspective of decision-support scientists, a critical first step in developing a PtDA is the “needs assessment”, which characterizes the sources of difficulty that the relevant patient population has with the specific decision situation, and their needs and preferences for a decision support intervention (with or without a PtDA) [19]. When an Internet-delivered PtDA is considered, a needs assessment is particularly complex, since it should also assess diverse individuals’ opinions about Internet-delivered clinical content, technology features, and implementation strategies [42,43,68]. Designers and developers of Internet-delivered PtDAs may seek to identify whether there are patterns in the preferred and effective features of such PtDAs, which then may be considered as standard attributes for acceptable quality.

Alternatively, investigators may seek to determine whether specific components of PtDAs delivered on the Internet should be targeted or tailored to the clinical context (e.g., preventive, acute, chronic, and end-of-life treatment decisions), the users’ socio-demographic characteristics (e.g., age, sex, race/ethnicity, culture, role in decision making), and/or the users’ geographical locations (e.g., urban/rural communities, international/national/regional sites). For example, is “value added” when patients are guided to either culturally-neutral or culturally-matched socially-derived experiential information and/or support? Furthermore, Internet-delivered PtDAs allow patients greater opportunity to customize or “self-tailor” the level of clinical information detail, engagement in deliberative steps, and viewing of socially-derived experiential information according to their personal deliberative styles. However, this customization may conflict with existing quality measures for standardized information provision and decision support across the sequence of steps in the decision-making process. Research is needed to identify approaches that provide high-quality decision support that meets general standards while simultaneously allowing for varying degrees of targeting, tailoring, customization, and personalization.

Population-level investigations may explore the potential added benefits and the possible risks of unintended harms generated by delivering PtDAs on the Internet, as well as the direct and indirect costs of developing, updating, and maintaining Internet-delivered PtDAs, compared to using other media [20,61]. Health services researchers may assess whether or not the delivery of PtDAs on the Internet fosters increased health equity for disadvantaged communities, chronic care, surrogate decision makers, isolated adults (e.g., individuals with mobility disabilities, home-bound older adults), and/or dispersed families [20,59,65,66]. Investigators in implementation science and translational research may seek to determine the best practices for using Internet-based PtDAs to deliver “the right support to the right patient at the right time” [69-71].

#### Research methods

While many research methods overlap across the relevant disciplinary perspectives, some diverge. Decision support researchers and health information technology researchers may include different steps in their design and evaluation process. Quality evaluators may use similar metrics, such as usability and acceptability, but may assess these in very different ways. Finally, they may focus on very different “high quality” outcomes.

For example, decision support research methods that arise from medical research models include: systematic reviews of the literature; needs assessments; prototype development; field-tests; effectiveness trials; and dissemination and implementation studies. During the field-testing of prototypes, usability and acceptability assessment may involve obtaining, from small groups of 10 – 30 patients, focus-group-based or questionnaire-based evaluations of the length, depth, level of detail, presence/freedom from bias, and interest generated by the clinical information and decision support steps [19,21]. Usability assessments may also include evaluations of the feasibility of using the new decision support tool in the flow of regular clinical care (e.g., before, during, or after a clinical consultation), or the feasibility of implementation across sites [20,63]. Other decision support outcomes may include measures of decisional conflict, preparation for decision making, decision quality, treatment choice, and adherence to chosen therapy [1,30,63].

Research methods that are specifically focused on Internet-delivered PtDAs could build on these medical research models by adding, early in the needs assessment and development phases, structured steps that specifically plan, analyze, design, and test the website itself [68]. These steps could include: assessing user characteristics and needs; developing narratives about target personas and key scenarios about site use; designing the content of the website; and incorporating iterative prototype-testing cycles [37-42]. For example, card sorting, wireframes, and task analyses may be used to structure the architecture of the content and to develop a prototype. Iterative cycles of prototype-testing with 3-5 users may include interactive achievement testing and heuristic evaluations that track eye movements when skimming/reading the content, and that note, for example, the number of clicks, the frequency of errors, and page-loading speeds. Usability metrics may include ease of learning (e.g., how quickly a new user can complete basic tasks using the site), efficiency, error frequency, memorability, and satisfaction [22,43]. Additional outcome measures may include continued website usage, accessibility for all types of users, and data privacy and security.

Therefore, in order to consider the quality (that is, the conceptual appropriateness, the validity, the sensitivity, etc.) of various research methods that could be used to develop, test, and implement Internet-delivered PtDAs, integrated conceptual frameworks and models are needed. These frameworks could inform the development of a shared language for identifying rigorous research practices and “high quality” outcome measures. There is a distinctive scientific challenge here: how best to integrate these various approaches in order to systematically develop a strong evidence base about the design, testing, and implementation of Internet-delivered PtDAs.

## Conclusion

At present, there is notable theoretical justification for delivering some or all of the components of a PtDA using the Internet. Several theories in psychology, education, communication, and implementation science support the value of the interactive, multimedia, and accessibility features of Internet-delivered PtDAs. However, there is currently a paucity of scientifically rigorous empirical studies investigating the role of Internet delivery for PtDAs, the usability of the user interfaces, and their effects in differing clinical, cultural, and decision-making contexts.

Accordingly, there is a rapidly expanding field of interdisciplinary research opportunities. In particular, studies are needed to assess the role of tailored information and support, the optimal uses of the unique features of the Internet, and the potential benefits and harms of socially-generated information about patient stories. Equity studies may also assess implementation strategies that include both paper-based PtDAs in medical centers and Internet-delivered PtDAs in rural communities. Ultimately, cost-effectiveness and cost-benefit analyses are needed to compare Internet-, video-, and paper-based strategies for dissemination, maintenance, and sustainability.

Finally, an interdisciplinary and internationally-representative approach is needed to identify best practices for a) needs assessments, b) user-centered development, c) usability assessments, d) field testing, d) clinical effectiveness, and e) broad and sustainable implementation strategies. As these gaps in the empirical evidence are addressed, the international scientific community may wish to revisit continually the criteria used to gauge the quality of Internet-delivered PtDAs.

## List of abbreviations used

IPDAS: International Patient Decision Aid Standards; PSA: prostate-specific antigen; PtDA: patient decision aid; RCT: randomized controlled trial; RE-AIM: Reach Effectiveness - Adoption Implementation Maintenance

## Competing interests

Linda C. Li has received research funding from Canadian Institutes of Health Research (CIHR), a national funding organization. Li has received funding to develop and evaluate two online patient decision aids for patients with rheumatoid arthritis.

Robert J. Volk has received research funding, travel support and honoraria from the Informed Medical Decisions Foundation, a not-for-profit (501 (c)3 ) private foundation (http://www.informedmedicaldecisions.org). The Foundation develops content for patient education programs. The Foundation has an arrangement with a for-profit company, Health Dialog, to co-produce these programs. The programs are used as part of the decision support and disease management services Health Dialog provides to consumers through health care organizations and employers.

All other authors have no competing interests to declare.

## Authors' contributions

AH led the conceptualization of the manuscript’s argument, led the updating and interpretation of the empirical evidence writing team, drafted the initial manuscript, led its critical revision for important intellectual content, and gave final approval of the version to be published.

RJV, AS, CS, LL, MH and GK made substantial contributions to the manuscript’s conceptualization, to the updating and interpretation of empirical evidence, and to the manuscript’s revisions, and gave final approval of the version to be published.

HLT made substantial contributions to the manuscript’s conceptualization, to the interpretation of empirical evidence, and to the manuscript’s revisions, and gave final approval of the version to be published.

## Supplementary Material

Additional file 1 **Table S1:** Design Characteristics of PtDAs Delivered on the Internet Click here for file

Additional file 2 **Table S2:** Emerging Studies of Internet-delivered PtDAs: Studies Conducted Since the Most Recent (2011) Cochrane Collaboration Review Click here for file

## Declarations

Publication of this supplement was partially funded by an unrestricted grant from the Informed Medical Decisions Foundation. The remainder costs were covered by Robert J. Volk. Administrative and editorial support for this supplement was also provided by The University of Texas MD Anderson Cancer Center.

This article has been published as part of *BMC Medical Informatics and Decision Making* Volume 13 Supplement 2, 2013: The International Patient Decision Aid Standards (IPDAS) Collaboration’s Quality Dimensions: Theoretical Rationales, Current Evidence, and Emerging Issues. The full contents of the supplement are available online at http://www.biomedcentral.com/bmcmedinformdecismak/supplements/13/S2.
