# Supplementary material for: Delivering patient decision aids on the Internet: definitions, theories, current evidence, and emerging research areas
Source: BMC Med Inform Decis Mak. 2013 Nov 29;13(Suppl 2):S13. doi: 10.1186/1472-6947-13-S2-S13 (PMC4043476; doi:10.1186/1472-6947-13-S2-S13)
Supplement: Additional file 1 — Table S1: Design Characteristics of PtDAs Delivered on the Internet [file 1472-6947-13-S2-S13-S1.pdf]

Table S1: Design Characteristics of PtDAs Delivered on the Internet [1-7]

**Linear vs. Open Format**	A PtDA website is considered linear if the content is presented in the same order every time and progression through the aid requires completion of each previous section (e.g., sequential pages). An open format allows the individual to navigate the website by selecting the type and order of information for viewing.
**Non-Interactive vs. Interactive**	PtDAs websites may range from non-interactive if the person viewing the site simply reads textual content, to minimally-interactive if they can select and view videos, to fully interactive if individuals can navigate the content and/or respond to interactive questions.
**Low/High-Engaging**	Across media, the content and guidance provided may elicit a spectrum of engagement from viewers (ranging from passive viewing to user-driven creation of personalized risk estimates and decision making summaries), depending on the match/mismatch of the design features used and the users' characteristics, decision support needs, and preferences.
**Implicit vs. Explicit Deliberative Guidance**	A PtDAs website may provide implicit decision support by describing the process of making a well-informed health care decision, or explicit decision support by actively leading the individual through the process of preparing to make their decision.
**Static vs. Dynamic**	A website is static if it is programmed to provide all viewers with the same design and content, and dynamic if it provides different information depending on what the individual selects.
**Text-heavy or Graphics-heavy**	PtDAs delivered on the Internet may present content using a variety of media, including text, figures, graphs, images, animations, audio, and videos. Mixed media refers to the use of more than one medium (e.g. text and photos), while multimedia refers specifically to the addition of audio (e.g. narration and videos). Rich media specifies the inclusion of interactive components, and hypermedia refers to interactive media that direct the presentation of information (e.g. hyperlinks that allow open navigation between pages).

**Anonymous, De-identified, and Identifiable**	PtDAs may be designed for anonymous use, where there is no way to connect the use of the decision aid with any particular person. De-identified means that if any data that might directly or indirectly identify a user is collected, it is obscured so there is only a very small chance the user could be determined. Identifiable means that the PtDA collects data that directly identifies the user. If identifiable information is collected, it is important to determine who will have access to that information. For example, password-protected accounts may be used to allow patients to revisit their information over time and/or clinicians to collect information directly into electronic health records. However, care must be used to ensure secure hosting and to avoid access by third party companies and/or Internet service providers.
**Tailored, Targeted, Customized, and Personalized**	Refers to PtDAs delivered on the Internet that may modify the content provided according to patients' characteristics, needs, and preferences in multiple ways. First, they can tailor clinical content based on an individual's unique characteristics or they can provide decision support targeted to a specific group of individuals. Sites may allow users to customize their experience by choosing which information and features are shown, or the site may personalize features based on how the user is using the site. For Internet-delivered PtDAs, these modifications may be made at several levels, based on socio-demographic (age, race, culture, etc.), clinical (risk profiles, health literacy, decisional capacity), decision making (capacity, deliberative style), or role (patient, caregiver, surrogate, legal proxy) characteristics.
**Accessibility of the Design**	Refers to the degree to which the PtDA is accessible to all people, particularly those with disabilities. PtDAs provided on the Internet may be designed to optimally work with assistive technologies, such as screen reading, screen magnifying, Braille, or speech recognition programs. Visual accessibility may be increased by providing larger font sizes, optimizing graphics for color-blindness, limiting flashing lights in videos, and by providing meaningful descriptions for images and links that can be read aloud by screen reading programs. Providing captions/subtitles for videos may increase audio accessibility. To increase motor accessibility, larger buttons, touch screens, or voice commands may be used. Providing options for high and low literacy levels may increase cognitive accessibility.

References

[1] Brownson RC, Colditz GA, Proctor EK: *Dissemination and implementation research in health: Translating science to practice.* New York: Oxford University Press; 2012.

[2] Nielsen J: *Designing Web Usability: The Practice of Simplicity***.** San Francisco: New Riders Publishing; 1999.

[3] Krug S. *Don't Make Me Think! A Common Sense Approach to Web Usability*. San Francisco: New Riders Publishing; 2006.

[4] Reddish J: *Letting Go of the Words: Writing Web Content that Works*. San Francisco: Morgan Kaufmann (Elsevier); 2007.

[5] Office of Disease Prevention and Health Promotion: *Health Literacy Online: A Guide to Writing and Designing Easy-to-Use Health Web Sites.* Washington DC: U.S. Department of Health and Human Services; 2010.

[6] Gilmour J: **Reducing disparities in the access and use of Internet health information.** *Int J Nurs Stud* 2007, **44**:1270-1278.

[7] Kreuter MW, Skinner CS: **Tailoring, what's in a name?** *Health Educ Res* 2000, **15**:1–4.
