# Supplementary material for: Delivering patient decision aids on the Internet: definitions, theories, current evidence, and emerging research areas
Source: BMC Med Inform Decis Mak. 2013 Nov 29;13(Suppl 2):S13. doi: 10.1186/1472-6947-13-S2-S13 (PMC4043476; doi:10.1186/1472-6947-13-S2-S13)
Supplement: Additional file 2 — Table S2: Emerging Studies of Internet-delivered PtDAs: Studies Conducted Since the Most Recent (2011) Cochrane Collaboration Review [file 1472-6947-13-S2-S13-S2.pdf]

Table S2. Emerging Studies of Internet-delivered PtDAs [1-8]**: Studies Conducted Since the Most Recent (2011) Cochrane Collaboration Review** [9]

**Author, Year, Reference Number**	**Clinical Context**	**Study Design**	**Primary Results**
Li, 2012, [1]	Rheumatoid arthritis	Development & field test, n = 15, of ANSWER interactive website PtDA	- Positive usability scores<br>- Acceptable viewing time<br>"Think Aloud" analysis yielded 3 themes:<br>- User engagement<br>- Information quality<br>- User interaction
Joseph-Williams, 2010, [2]	PSA screening	Observational study, n = 82, usage data examined for associations between Prosdex usage and components of informed decision making	Increased Prosdex usage was significantly associated with:<br>- Increased knowledge<br>- Decreased favorable attitude about PSA testing<br>- Reduced intention to undergo PSA testing
Evans, 2010, [3]	PSA screening	RCT, n = 514, randomized to 4 groups: viewing Prosdex website in location of their choice vs. paper version, with baseline questionnaire or without	Compared to control, viewing Prosdex:<br>- Increased knowledge scores<br>- Decreased favorable attitude about PSA screening<br>- Decreased screening<br>- Decreased decisional conflict<br>Prosdex outcomes did not differ significantly with paper version
Miller, 2011, [4]	Colorectal cancer	RCT, n = 264, randomized to CHOICE, an Internet-delivered video, versus control video	CHOICE video group reported:<br>- Increased post-intervention preference<br>- Increased readiness to receive screening
Simon, 2012, [5]	Depression & acute low back pain	RCT, n = 131, randomized to tailored Patient Dialogue website or static patient information	Patient Dialogue group reported:<br>- Lower decisional conflict scores<br>- Higher preparation for decision making scores

Hoffman, 2012, [6]	Knee osteoarthritis	Development and field test, n = 126, of interactive, step-wise, tailored Internet PtDA, compared to usual care (with video PtDA)	Development model integrated decision support and informatics methods<br>Internet PtDA group reported:<br>- Knowledge scores and usability scores above usual care<br>- 100% preference to view PtDA on home/public computer<br>- Higher preparation and self-efficacy scores<br>- Lower decisional conflict scores
Sivell, 2012, [7]	Breast cancer surgery	Observational cohort study, n = 54, evaluating use of BresDex, based on collected web-log data of components accessed and time spent, overall and on each component	- Using BresDex increased readiness to make surgery decision<br>- More time spent on BresDex was associated with stronger intentions to have surgery and lower pre-intervention readiness to make a surgery decision<br>- Surgery intentions and choices were significantly related<br>- Majority of participants went on to have surgery
Durand, 2012, [8]	Amniocentesis	Development of two deliberation components for AmnioDex, a graphic, interactive, web-based PtDA based on "Take the Best" heuristics and integration algorithm. Evaluation, n = 48, by researchers, healthcare providers, and pregnant women.	Researchers reported that the tools:<br>- Were useful in facilitating decision making<br>- Need simple instructions, clear layouts<br>Health-care providers expressed concerns regarding the usability and clarity of the tools<br>Pregnant women found the tools useful in:<br>- Weighing pros and cons<br>- Clarifying their thoughts<br>- Visualizing their decision efforts<br>- Potentially inappropriate for this difficult, emotional decision

References

[1] Li LC, Townsend AF, Badley EM: **Self-management interventions in the digital age: New approaches to support people with rheumatologic conditions**. *Best Pract Res Clin Rheumatol* 2012, **26**:321-333.

[2] Joseph-Williams N, Evans R, Edwards A, Newcombe RG, Wright P, Grol R, Elwyn G: **Supporting informed decision making online in 20 minutes: an observational web-log study of a PSA test decision aid**. *J Med Internet Res* 2010, **12**:e15.

[3] Evans R, Joseph-Williams N, Edwards A, Newcombe RG, Wright P, Kinnersley P, Griffiths J, Jones M, Williams J, Grol R *et al*: **Supporting informed decision making for prostate specific antigen (PSA) testing on the web: an online randomized controlled trial**. *J Med Internet Res* 2010, **12**:e27.

[4] Miller DP, Jr., Spangler JG, Case LD, Goff DC, Jr., Singh S, Pignone MP: **Effectiveness of a web-based colorectal cancer screening patient decision aid: a randomized controlled trial in a mixed-literacy population**. *Am J Prev Med* 2011, **40**:608-615.

[5] Simon D, Kriston L, von Wolff A, Buchholz A, Vietor C, Hecke T, Loh A, Zenker M, Weiss M, Harter M: **Effectiveness of a web-based, individually tailored decision aid for depression or acute low back pain: a randomized controlled trial**. *Patient Educ Couns* 2012, **87**:360-368.

[6] Hoffman AS, Llewellyn-Thomas HA, Tosteson ANA, O'Connor AM, Volk RJ, Tomek I, Bartels SJ, Andrews SA: **Development of an Internet-based patients' decision aid for surgical versus nonsurgical management of knee osteoarthritis**. [abstract] The 34th Annual Meeting of the Society for Medical Decision Making; 2012. [http://smdm.confex.com/smdm/2012az/webprogram/Session1601.html]

[7] Sivell S, Edwards A, Manstead AS, Reed MW, Caldon L, Collins K, Clements A, Elwyn G: **Increasing readiness to decide and strengthening behavioral intentions: evaluating the impact of a web-based patient decision aid for breast cancer treatment options (BresDex: www.bresdex.com)**. *Patient Educ Couns* 2012, **88**:209-217.

[8] Durand MA, Wegwarth O, Boivin J, Elwyn G: **Design and usability of heuristic-based deliberation tools for women facing amniocentesis**. *Health Expect* 2012, **15**:32-48.

[9] Stacey D, Bennet CL, Barry MJ, Col NF, Eden KB, Holmes-Rovner M, Llewellyn-Thomas HA, Lyddiatt A, Légaré F, Thomspon R: **Decision aids for people facing health treatment or screening decisions**. *Cochrane Database of Syst Rev* 2011.
